# Assessment of Serum sRANKL, sRANKL/OPG Ratio, and Other Bone Turnover Markers with the Estimated 10-Year Risk of Major and Hip Osteoporotic Fractures in Rheumatoid Arthritis: A Cross-Sectional Study

**DOI:** 10.1155/2021/5567666

**Published:** 2021-08-30

**Authors:** C. A. Nava-Valdivia, J. M. Ponce-Guarneros, A. M. Saldaña-Cruz, E. G. Corona-Sanchez, M. Ramirez-Villafaña, E. E. Perez-Guerrero, J. D. Murillo-Saich, B. Contreras-Haro, M. L. Vazquez-Villegas, F. Gonzalez-Ponce, D. Bonilla-Lara, H. Jacobo-Cuevas, J. D. Centeno-Valadez, G. Echeverria-Gonzalez, S. Cerpa-Cruz, M. F. Alcaraz-Lopez, E. G. Cardona-Muñoz, M. Salazar-Paramo, L. Gonzalez-Lopez, J. I. Gamez-Nava

**Affiliations:** ^1^Departamento de Microbiología y Patología, Centro Universitario de Ciencias de la Salud, Universidad de Guadalajara, 44340 Guadalajara, JAL, Mexico; ^2^Unidad Médica Familiar 97, Instituto Mexicano del Seguro Social (IMSS), 46470 Magdalena, JAL, Mexico; ^3^Instituto de Terapéutica Experimental y Clínica (INTEC), Departamento de Fisiología, Centro Universitario de Ciencias de la Salud, Universidad de Guadalajara, 44340 Guadalajara, JAL, Mexico; ^4^Departamento de Fisiología, Centro Universitario de Ciencias de la Salud, Universidad de Guadalajara, 44340 Guadalajara, JAL, Mexico; ^5^Instituto de Investigación en Reumatología y del Sistema Músculo Esquelético, Centro Universitario de Ciencias de la Salud, Universidad de Guadalajara, 44340 Guadalajara, JAL, Mexico; ^6^Instituto de Investigación en Ciencias Biomédicas (IICB), Centro Universitario de Ciencias de la Salud, Universidad de Guadalajara, 44340 Guadalajara, JAL, Mexico; ^7^Department of Medicine, School of Medicine, University of California, San Diego, 9500 Gilman Drive, San Diego, CA 92093, USA; ^8^Departamento de Ciencias Biomédicas, Centro Universitario de Tonalá, Universidad de Guadalajara, 45425 Tonalá, JAL, Mexico; ^9^Programa de Doctorado en Ciencias de la Salud Pública, Departamento de Salud Pública, Centro Universitario de Ciencias de la Salud, Universidad de Guadalajara, 44340 Guadalajara, JAL, Mexico; ^10^Programa de Doctorado en Farmacología, Departamento de Fisiología, Centro Universitario de Ciencias de la Salud, Universidad de Guadalajara, 44340 Guadalajara, JAL, Mexico; ^11^Departamento de Reumatología, Hospital de Especialidades, Centro Médico Nacional de Occidente, Instituto Mexicano del Seguro Social, 44329 Guadalajara, JAL, Mexico; ^12^División de Reumatología, Hospital Civil de Guadalajara “Fray Antonio Alcalde”, 44280, Guadalajara, JAL, Mexico; ^13^Departamento de Medicina Interna-Reumatología, Hospital General Regional 46, IMSS, 44910 Guadalajara, JAL, Mexico; ^14^Departamento de Medicina Interna-Reumatología, Hospital General Regional 110, IMSS, 44716, Guadalajara, JAL, Mexico; ^15^Unidad de Investigación Biomédica 02, División de Investigación, Hospital de Especialidades, Centro Médico Nacional de Occidente (CMNO), Instituto Mexicano del Seguro Social (IMSS), 44340 Guadalajara, JAL, Mexico

## Abstract

**Background:**

Fracture risk assessment tool (FRAX) index was developed for estimating of the 10-year risk of major or hip osteoporotic fracture. To date, there is insufficient information regarding the correlation between FRAX and serum bone turnover markers (BTMs), such as soluble ligand of receptor activator of nuclear factor-*κ*B (sRANKL), osteoprotegerin (OPG), and other molecules related with secondary osteoporosis in rheumatoid arthritis (RA). Therefore, this study is aimed at assessing the correlation between the FRAX and serum levels of sRANKL, OPG, sRANKL/OPG ratio, Dickkopf-1 (DKK-1), and sclerostin (SOST) in RA.

**Methods:**

Cross-sectional study included 156 postmenopausal women with RA. Bone mineral density (BMD) was measured at lumbar spine (L1-L4) and total hip using dual-energy X-ray absorptiometry (DXA). RA patients were divided into (A) RA + osteoporosis and (B) RA without osteoporosis. FRAX scores were calculated including the total hip BMD. Serum sRANKL, OPG, DKK-1, and SOST levels were measured by ELISA. Pearson tests were used for assessing the correlation between serum levels of these molecules and FRAX scores in RA.

**Results:**

The RA + osteoporosis group had elevated sRANKL levels (*p* = 0.005), higher sRANKL/OPG ratio (*p* = 0.017), decreased DKK-1 (*p* = 0.028), and lower SOST levels (*p* < 0.001). Low total hip BMD correlated with high sRANKL (*p* = 0.001) and sRANKL/OPG ratio (*p* = 0.005). Total hip and lumbar spine BMD correlated with DKK-1 (*p* = 0.009 and *p* = 0.05, respectively) and SOST levels (*p* < 0.001 and *p* < 0.001, respectively). Higher sRANKL levels and sRANKL/OPG ratio correlated with estimated 10-year risk of a major osteoporotic fractures (*p* = 0.003 and *p* = 0.003, respectively) and hip fracture (*p* = 0.002 and *p* = 0.006, respectively). High serum SOST levels were associated with a low estimated 10-year risk of a major osteoporotic fracture (*p* = 0.003) and hip fracture (*p* = 0.009).

**Conclusion:**

High sRANKL levels and sRANKL/OPG ratio can be useful to detect a subgroup of RA patients who has an increased 10-year risk of major and hip osteoporotic fractures.

## 1. Introduction

Rheumatoid arthritis (RA) is a chronic systemic autoimmune disease characterized by an imbalance of cellular, proinflammatory mediators, and autoantibodies that lead to synovial joint inflammation and the destruction of cartilage and bone in severe cases [1, 2]. The overall progressive bone loss has been considered a risk factor for osteoporosis in patients with RA due to inherent factors related to the disease [3]. Osteoporosis is highly prevalent in RA patients (38.6%) [4]. In Mexican women with RA, the prevalence of osteoporosis is around 24% [5]. The current gold standard for diagnosis of osteoporosis measures of bone mineral density (BMD) by dual-energy X-ray absorptiometry (DXA) [6]; therefore, the determination of BMD is mandatory in patients with RA.

However, in addition to measuring BMD, other risk factors of osteoporosis have been incorporated into risk assessment tools to predict the development of osteoporotic fractures. One of the main tools developed for this purpose is the fracture risk assessment tool (FRAX), which is an algorithm developed to estimate the 10-year risk of major osteoporotic fractures or the 10-year risk of a hip fracture. To calculate FRAX scores, total hip BMD values can be alternatively introduced in the formula [7]. Studies report a significant increase in the risk of fracture in patients with RA compared to the healthy population [8]. Furthermore, several studies have evaluated the relevance of the FRAX score in RA patients. Phuan-Udom et al. found an association between the FRAX score with disease duration, disease activity, and functional disability in RA patients [9]. Cheng et al. observed that the10-year probability of fracture increase in anti-CCP positive patients with RA [10].

Whereas several bone turnover markers (BTMs) have been used in nonrheumatic population to assess the risk of osteoporosis independently of BMD values by DXA [6, 11], one of the advantages of BTMs is that they measure the rate of bone remodeling changes [12]. Furthermore, serum biomarkers of bone resorption such as soluble ligand of receptor activator of nuclear factor-*κ*B (sRANKL), osteoprotegerin (OPG), and sRANKL/OPG ratio have been previously investigated in RA patients with osteoporosis [13, 14]. Nonetheless, the assessment of BTMs in RA patients could contribute to a better understanding of the most significant biomarkers associated with the development of osteoporosis. Among these markers, some authors have found abnormal serum levels of Dickkopf-1 (DKK-1) [15] and sclerostin (SOST) [16] in RA patients with low BMD. However, these studies did not include in their patients the assessment of serum sRANKL and OPG levels. There is a lack of studies that assess whether the serum levels of these bone markers could correlate or not with FRAX scores in RA patients to establish early diagnosis and personalized patient management.

To date, there is some information regarding the relation between several bone turnover markers and osteoporosis in RA; however, there is a lack of studies assessing whether the serum levels of these bone markers could correlate or not with the FRAX score in RA patients. Therefore, this study is aimed at assessing the correlation between FRAX scores for the estimated 10-year risk of a major and hip osteoporotic fractures and the serum levels of sRANKL, OPG, sRANKL/(OPG) ratio, Dickkopf-1 (DKK-1), and sclerostin (SOST) in RA patients.

## 2. Materials and Methods

### 2.1. Study Design

Cross-sectional.

### 2.2. Clinical Setting

RA patients referred to undergo bone densitometry at a tertiary care center were recruited from a public hospital of the Mexican Institute of Social Security (*Hospital de Especialidades*, *Centro Medico Nacional de Occidente*, *Instituto Mexicano del Seguro Social*) in Guadalajara, Mexico.

### 2.3. Inclusion and Exclusion Criteria

We included 156 consecutive Mexican postmenopausal women with RA who met the following inclusion criteria: (1) female gender, (2) postmenopausal diagnosis, and (3) established RA according to the American College of Rheumatology (ACR, 1987) criteria [17]. Patients were excluded if they were receiving antiresorptive drugs, a daily glucocorticoid dose ≥ 15 mg, or treatment with biologic disease-modifying antirheumatic drugs (b-DMARDs). We also excluded patients with active acute infections at the time of the study, overlapping syndrome, chronic infections including hepatitis B and C or the human immunodeficiency virus, patients with chronic renal failure (creatinine > 1.5 mg/dL), transaminases > 2 − fold the upper limit of normal values, cancer, or with an overlap syndrome.

### 2.4. Clinical Evaluations

Three trained researchers used a structured questionnaire to evaluate risk factors for osteoporosis. Current clinical and therapeutic RA evaluations were also conducted. At the same visit, a physical examination and a clinical evaluation of disease activity with the validated Disease Activity Score of 28 joints using the erythrocyte sedimentation rate (DAS28-ESR) were performed [18]. The assessment of functional capacity was carried out using the validated Spanish version of the Health Assessment Questionnaire Disability Index (HAQ-Di) [19].

### 2.5. Bone Mineral Density (BMD) Assessment

Dual-energy X-ray absorptiometry (DXA) was used to quantify bone mineral density (BMD) (g/cm^2^) at the lumbar spine (L1-L4) and total hip region using a Lunar 2000 Prodigy Advance DXA scanner (GE Medical Systems Lunar software 8.8, Madison WI, USA). BMD of the lumbar spine and total hip was classified according to the International Society for Clinical Densitometry guidelines [6]. Osteoporosis was diagnosed in postmenopausal women using *T*-scores when the BMD results of the lumbar spine or total hip showed a decrease below <-2.5 standard deviations (SD). Patients were classified into two groups: (A) RA + osteoporosis (BMD at the total hip or lumbar spine equal or below -2.5 standard deviations of the *T*-score) and (B) RA without osteoporosis (BMD at the total hip or lumbar spine above -2.5 standard deviations of the *T*-score).

### 2.6. Fracture Risk Assessment Tool (FRAX)

The estimated 10-year risk of major and hip osteoporotic fractures in RA patients was determined using the FRAX algorithm calculator for Mexico (https://www.sheffield.ac.uk/FRAX/tool.aspx?lang=sp). The FRAX algorithm has a set of clinical risk factors for osteoporotic fractures that include sex, age, BMI, history of fragility fracture, current smoking and alcohol use, glucocorticoid use, and the presence of rheumatoid arthritis. We included the results of total hip BMD in gr/cm^2^ for the calculation [7]. FRAX scores for the 10-year probability of major osteoporotic fractures and 10-year probability of hip fracture were computed using the total hip BMD.

### 2.7. Laboratory Assessment

Erythrocyte sedimentation rate (ESR) in mm/h was determined using the Westergren method. Rheumatoid factor (RF) concentrations were quantified by nephelometry. Second-generation anticyclic-citrullinated peptide (anti-CCP2) was quantified by enzyme-linked immunosorbent assay (ELISA) (Euroimmun AG, Luebeck, Germany. Sensitivity 0.2 RU/mL). Anti-CCP2 positivity was considered at ≥5 RU/mL. Autoantibodies against mutated citrullinated vimentin (anti-MCV) were quantified by ELISA with a commercial kit (Orgentec Diagnostika GmbH, Mainz, Germany; assay sensitivity of 1.0 UI/mL), and a value of ≥20 UI/mL was considered positive. Serum levels of tumor necrosis factor-alpha (TNF-*α*) were quantified by ELISA using a commercial kit (R&D Systems Inc., Minneapolis, MN, USA; assay sensitivity of 5.5 pg/mL).

### 2.8. Measurement of Bone Turnover Markers

Serum levels of sRANKL, OPG, DKK-1, SOST, and other markers were quantified by ELISA using commercial kits: sRANKL (Biovendor Laboratorni Medicina A. S., Brno, Czech Republic; assay sensitivity of 0.4 pmol/L), OPG (RayBiotech Inc., Norcross, GA, USA; assay sensitivity of 1.0 pg/mL), DKK-1 (R&D Systems Inc., Minneapolis, MN, USA; assay sensitivity of 0.94 pg/mL), SOST (R&D Systems Inc., Minneapolis, MN, USA; assay sensitivity of 0.37 pg/mL), OC (osteocalcin) (Quidel Corporation, San Diego, CA, USA; assay sensitivity of 0.47 ng/mL), and CTSK (cathepsin k) (MyBiosource, Inc., San Diego, CA, USA; assay sensitivity of 0.1 ng/mL). All procedures were performed according to manufacturer's recommendations. sRANKL/OPG ratio was also calculated. All measurements were performed by the same researchers who were blind to the clinical characteristics of patients to avoid measurement biases.

### 2.9. Statistical Analysis

Qualitative variables were expressed as frequencies (%) and quantitative variables as means ± standard deviations (SD). The comparisons of proportions between study groups were analysed using Chi-square tests (or Fisher's exact tests, if required). The comparison of means between the study groups RA + osteoporosis and RA without osteoporosis groups was performed using the Student's unpaired *t*-test. Correlations between sRANKL, OPG, DKK-1, SOST, and other BTMs with clinical and laboratory variables were calculated using Pearson correlation tests. Statistical significance was set at a *p* value ≤ 0.05. All statistical analyses were performed using R version 4.0.0 [20] and GraphPad Prism software version 7.00 (San Diego, California, USA).

## 3. Results

This study included 156 postmenopausal women with RA, of which, 49.4% had osteoporosis. The prevalence of osteoporosis in total hip was 40.4% (*n* = 63), and the frequency of osteoporosis at the lumbar spine was 35.3% (*n* = 55) (data not shown).

Of 125 RA patients (80%), who received pharmacological treatment with conventional synthetic-disease-modifying antirheumatic drugs (cs-DMARDs), of which, 36.5% (*n* = 57) were treated with a monotherapy scheme, and 43.6% (*n* = 68) received a combination of two or more cs-DMARDs. Methotrexate was the cs-DMARD most frequently prescribed (46.2%, *n* = 72), followed by leflunomide (30.1%, *n* = 47), sulfasalazine (28.8%, *n* = 45), chloroquine (14.1%, *n* = 22), and azathioprine (13.5%, *n* = 21). There were no statistical differences in the type of cs-DMARDs used between groups (data not shown).

**Figure 1:**
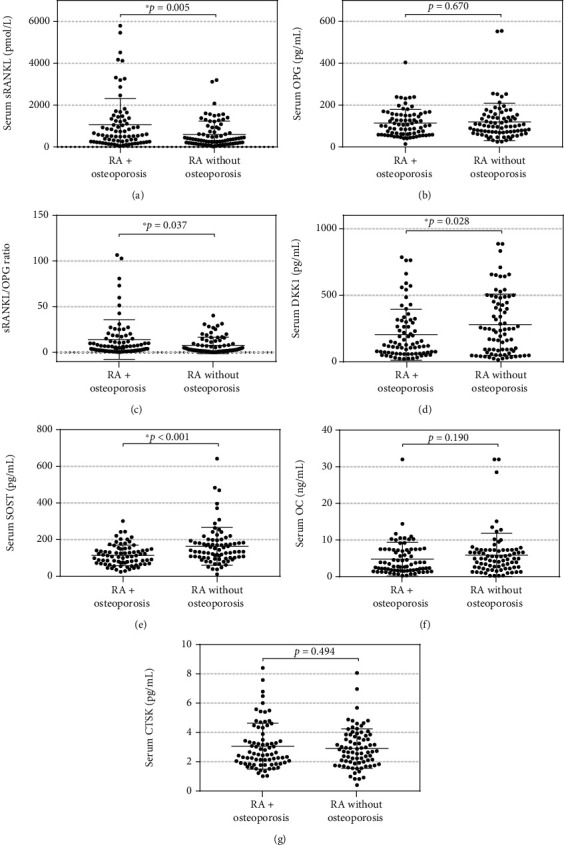
Comparison between the serum levels of molecules associated with bone remodeling between the RA + osteoporosis and RA without osteoporosis groups. *p* values were obtained using Student's *t*-test. Figure 1 shows the comparisons of the following serum markers: (a) sRANKL levels; (b) OPG levels; (c) sRANKL/OPG ratio; (d) DKK-1 levels; (e) SOST levels; (f) osteocalcin levels; (g) cathepsin k levels.

Table 1 compares the clinical characteristics between the RA + osteoporosis group (*n* = 77) versus the RA without osteoporosis group (*n* = 79). The RA + osteoporosis group had a higher age range (63 vs. 56 years, *p* < 0.001), lower BMI (26.6 vs. 29.2 kg/m^2^, *p* < 0.001), longer period from the onset of menopause (17 vs. 10 years, *p* < 0.001), and higher ESR levels (28 vs. 23 mm/h, *p* = 0.006). The RA + osteoporosis group had a similar frequency of anti-CCP positivity (58.4% vs. 59.5%, *p* = 1.0). A nonsignificant trend of increased RA disease duration was observed in patients with RA + osteoporosis (14 vs. 11 years, *p* = 0.058) and a higher frequency of anti-MCV antibodies (66.2% vs. 50.6%, *p* = 0.053). Patients with RA + osteoporosis had higher sRANKL levels (1063.7 vs. 602.3 pmol/L, *p* = 0.005), higher sRANKL/OPG ratio (14.1 vs. 7.6, *p* = 0.017), lower serum concentrations of DKK-1 (204.1 vs. 279.4 pg/mL, *p* = 0.028), and decreased serum SOST levels compared with RA patients without osteoporosis (114.1 vs. 163.5 pg/mL, *p* < 0.001). There were no significant statistical differences between both study groups in the other biomarkers (Figure 1).

Table 2 shows the correlations between the total hip and lumbar spine BMD (in g/cm^2^) with bone marker levels and other features. Total hip BMD negatively correlated with age (*p* < 0.001), RA disease duration (*p* = 0.003), the duration of menopause (*p* < 0.001), ESR (*p* = 0.003), sRANKL levels (*p* = 0.001), and the sRANKL/OPG ratio (*p* = 0.005), while increased total hip BMD correlated with BMI (*p* = 0.001), higher DKK-1 (*p* = 0.009), and SOST serum levels (*p* < 0.001). Lumbar spine BMD negatively correlated with age (*p* < 0.001) and duration of menopause (*p* < 0.001). The increased lumbar spine BMD correlated with an elevated BMI (*p* = 0.025), high DKK-1 (*p* = 0.050), and SOST serum levels (*p* < 0.001).

The sRANKL serum levels correlated positively with long RA disease duration (*r* = 0.172, *p* = 0.031), ESR (*r* = 0.257, *p* = 0.001), and anti-MCV levels (*r* = 0.404, *p* < 0.001). The OPG had a positive correlation with CTSK serum levels (*r* = 0.166, *p* = 0.038). The sRANKL/OPG ratio correlated positively with ESR (*r* = 0.209, *p* = 0.009) and anti-CCP titters (*r* = −0.359, *p* < 0.001). DKK-1 levels correlated with SOST levels (*r* = 0.181, *p* = 0.024) and ESR (*r* = 0.200, *p* = 0.012), whereas DKK-1 had a negative correlation with age (*r* = −0.255, *p* = 0.001). SOST levels were negatively correlated with anti-CCP concentrations (*r* = −0.213, *p* = 0.008), age (*r* = −0.177, *p* = 0.027), and the duration of menopause (*r* = −0.165, *p* = 0.040). Serum CTSK levels correlated with OPG levels (*r* = 0.166, *p* = 0.038) and the glucocorticoid dose administered per day (*r* = 0.225, *p* = 0.005). OC levels did not have significant correlation with other variables (data not shown in tables).

The prevalence of RA patients with high risk of major osteoporotic fracture (FRAX score > 20%) was 12.8% (*n* = 20), whereas 27.6% (*n* = 43) had a moderate risk of major osteoporotic fracture (FRAX score 10-20%), and 59.6% (*n* = 93) of the RA patients had a low risk of a major osteoporotic fracture (FRAX score < 10%). Also, the prevalence of RA patients with high risk of hip fracture (FRAX score > 10%) was 10.9% (*n* = 17); 23.7% (*n* = 37) had a moderate risk of hip fracture (FRAX score 3-10%), and 65.4% (*n* = 102) had a low risk of a hip fracture (FRAX score < 3%) (Data not shown).

**Figure 2:**
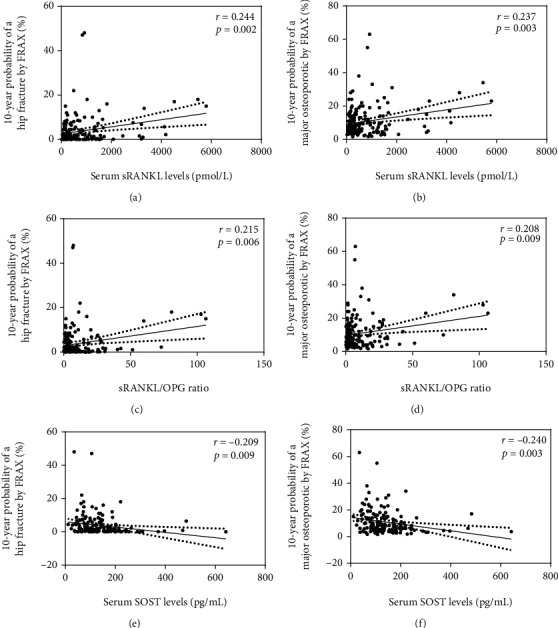
Correlations between the 10-year risk of major osteoporotic fractures and hip fractures (FRAX score) with serum sRANKL levels, sRANKL/OPG ratio, and serum SOST levels in RA patients. Figure 2 shows the correlations of serum sRANKL levels with the 10-year probability of hip fracture (a) and the 10-year probability of major osteoporotic fracture (b), the sRANKL/OPG ratio with 10-year probability of hip fracture (c) and 10-year probability of major osteoporotic fracture (d), and serum SOST levels with 10-year probability of hip fracture (e) and 10-year probability of major osteoporotic fracture (f).

Table 3 shows the correlations between the clinical variables, laboratory features, serum bone biomarker concentrations, and the 10-year risk of hip and major osteoporotic fractures according to the FRAX score. Serum sRANKL levels correlated with a higher 10-year risk of major osteoporotic fractures (*p* = 0.003) and a higher 10-year risk of hip fractures (*p* = 0.002). Likewise, the sRANKL/OPG ratio correlated with a higher 10-year risk of major osteoporotic fractures (*p* = 0.009) and higher risk to hip fractures (*p* = 0.006). In addition, increased serum SOST levels correlated negatively with the 10-year risk assessment of major osteoporotic fractures (*p* = 0.003) and with the 10-year risk of hip fractures (*p* = 0.009) (Figure 2).

## 4. Discussion

We identified a significant increase of sRANKL levels and the sRANKL/OPG ratio in RA patients with osteoporosis. Furthermore, sRANKL levels and sRANKL/OPG ratio were both correlated with a low total hip BMD and higher FRAX scores, indicating that these patients had an increase 10-year risk of major and hip osteoporotic fractures. Instead, higher DKK-1 and SOST levels were identified in RA patients without osteoporosis patients, correlating with high total hip and lumbar spine BMD, as well as with a decreased of major and hip osteoporotic fracture assessed by FRAX.

In the present study, the prevalence of osteoporosis in RA was 49.4%, in total hip 40.4%, and in lumbar spine 35.3%. This prevalence was similar in different studies according to a meta-analysis by Wang et al. where the pooled prevalence of osteoporosis in the lumbar spine was 32.9% (range from 27.7% to 38.1%) and 21.7% in the femur (range from 10.6% to 32.8%) [4]. The prevalence of central osteoporosis in our study was similar to a study by Lee et al. in Korean RA patients whose osteoporosis prevalence was 46.8% [21].

We observed a 12.8% of high risk of major osteoporotic fractures and 10.9% of high risk of hip fracture in RA patients. These findings were similar to those reported by Phuan-Udom et al., in RA patients [9]. This result confirms the high prevalence of high risk of osteoporotic fractures in RA.

RANKL is a type II membrane protein, expressed by osteoblasts, stromal cells, and T lymphocytes [22]. This molecule is secreted to the extracellular medium and binds to RANK, which is the natural receptor present in immature osteoclasts. The link RANKL to RANK unchains the maturation and activation of osteoclasts required for bone resorption [23]. An overexpression of RANK and RANKL developed by the overactivation of T cells leads to the increased production of RANKL released to the circulating blood that has been observed in RA patients. Additionally, RANKL is released into the synovium joint of these patients and binds to the RANK receptor in osteoclasts, which accelerates the bone resorption process. Therefore, an increase in the link between RANKL with RANK contributes to a low BMD in RA patients [23]. On the other hand, OPG is a soluble protein secreted by osteoblasts that exerts an opposite effect, acting as a protective molecule for bone resorption [24]. OPG is a decoy receptor of RANKL that inhibits the maturation and activation of osteoclasts through competition between the binding of RANKL and RANK [22]. In osteoporosis associated with RA, we did not observe an increase in OPG levels to protect BMD. It has been observed that some risk factors of osteoporosis increase the production of proinflammatory cytokines, particularly IL-1*β* and IL-6, and can decrease OPG serum levels [25]. All these factors are present in RA patients, leading to the high prevalence of osteoporosis found in our study.

When we investigated the association between sRANKL levels and osteoporosis in RA patients, we found in the correlation analysis, a correlation between high sRANKL levels with a low total hip BMD. This finding was similar to those obtained in previous studies on a postmenopausal nonrheumatic population and RA patients [14, 26]. Nabipour et al. identified that a low femoral neck BMD was associated with high sRANKL levels [26]. On the other side, Oelzner et al. observed that a low femoral neck BMD was associated with high sRANKL levels in RA patients [14]. Similarly, Xu et al. also identified an association between high sRANKL levels and low femoral neck BMD in RA patients compared to controls [13].

In contrast, Liu et al. did not observe association between sRANKL with osteoporosis among postmenopausal nonrheumatic patients [27]. In this study, we did not observe an association between low serum OPG levels and osteoporosis in RA patients. These findings support the results by Oelzner et al., where no association was found between OPG levels and the femoral neck or lumbar spine BMD [14]. On the other hand, Xu et al. observed an association between high OPG levels and an increased femoral neck and lumbar spine BMD [13].

We observed that RA patients with osteoporosis had a significant increase of the sRANKL/OPG ratio. These findings are in line with those of Nabipour et al. who also observed that a low femoral neck BMD was associated with an increase of the sRANKL/OPG ratio [26].

We found a correlation between FRAX scores for the10-year probability of a major osteoporotic fractures and 10-year probability of a hip fracture with higher serum levels of sRANKL and sRANKL/OPG ratio. These findings agree with our hypothesis that sRANKL and sRANKL/OPG ratio are linked to an increased risk of major osteoporotic and hip fracture. To the best of our knowledge, there are no previous studies published in the literature evaluating the correlation between FRAX score and sRANKL or OPG levels in RA. Mykhailovska et al. reported in a group of patients with coronary artery disease an association between the serum OPG levels with an increase in the FRAX score for the risk of radial osteoporotic fracture [28]. We did not observe any correlation between serum OPG levels with the FRAX scores.

DKK-1 is a molecule expressed by osteoblasts that inhibits the Wingless tail (Wnt)/*β*-catenin pathway, a relevant system that participates in bone remodeling and plays a key role in the cell signaling, proliferation, and differentiation of preosteoblastic cells, and inhibits their apoptosis [29–31]. DKK-1 is recognized as an inhibitor of the Wnt/*β*-catenin pathway. DKK-1 is also secreted by synovial fibroblasts, chondrocytes, and mature osteocytes [32–34]. Under normal conditions, an increase in DKK-1 is related to a reduction in bone formation [35].

On other hand, SOST is a glycoprotein expressed and secreted by mature osteocytes and joint chondrocytes. SOST binds to the LRP5/6 receptor and inhibits signaling of the WNT-*β* catenin pathway, causing a reduction in bone formation [36, 37]. Among the factors involved in the serum level increase of these molecules, the expression of SOST in fibroblast-like synoviocytes in RA and the poor relationship between gene expression and SOST serum concentrations have been reported [38, 39]. In animal models of adjuvant-induced arthritis, SOST and DKK-1 can be overexpressed initially, and later, a decrease in bone mass formation is observed; in spite of this, SOST and DKK-1 can return to normal values [40].

We investigated whether DKK-1 and SOST serum levels are biomarkers of osteoporosis in RA patients. Contrary to our primary hypothesis, we found that high levels of DKK-1 were associated with high total hip and lumbar spine BMD, and DKK-1 values were higher in patients without osteoporosis. Similar to our findings, Ueland et al. observed that cortical bone matrix levels of DKK1 and SOST were correlated with bone mass in postmenopausal women without rheumatic disease [41]. These findings reflect the complexity of the dynamic behaviour of this serum marker.

In the correlation analysis, we found that DKK-1 and SOST levels correlated with an increase in total hip BMD. RA patients with osteoporosis had higher DKK-1 and SOST levels than RA patients without osteoporosis. These findings are in contrast to those observed by Rossini et al. who found increased in serum DKK-1 levels correlated with a low total hip BMD [15]. In postmenopausal patients without rheumatic disease, discrepant results of the relationship between DKK-1 and BMD were also observed. Ardawi et al. reported a negative correlation between serum DKK-1 levels and BMD of the lumbar spine, femoral neck, and total hip in a nonrheumatic population [42]. Similarly, Coulson et al. described an association between DKK-1 levels and low BMD in people without inflammatory rheumatic disorders [43]. The results of our study differ from those described by Rossini et al. because we identified a positive correlation between DKK-1 concentrations and an increase in total hip BMD. Different reasons could explain these differences. Particularly, we avoided the inclusion of potential confounders, including drugs that could influence BMD. In contrast, in Rossini's study, the use of biologic agents and bisphosphonates was 30% and 44%, respectively [15].

We identified that RA patients with osteoporosis had increased SOST levels. Nevertheless, there are a few studies that have examined the relationship between SOST levels and BMD in RA patients. Therefore, we compared our findings mainly with those observed in nonrheumatic population. Garnero et al., in postmenopausal osteoporosis without rheumatic inflammatory disorders, identified that high SOST levels correlated with an increase in total hip and lumbar spine BMD [37]. However, other authors have observed different results. Ardawi et al. reported a negative correlation between serum SOST levels and BMD for the lumbar spine, femoral neck, and total hip in nonrheumatic population [42]. Some authors have also found an association between increased SOST levels and low BMD in postmenopausal patients without rheumatic disorders [37, 44]. Similar to our findings, Paccou et al. observed a positive correlation between serum SOST levels and the lumbar spine BMD [16]. In addition, Paccou et al. and Arasu et al. hypothesized that the correlation observed between SOST levels and an increase in BMD could be associated with an increase in the number of osteocytes producing circulating SOST [16, 44]. Meanwhile, Reppe et al. reported a correlation between SOST levels, and an increase in BMD could be associated with an increased methylation in the *SOST* gene promoter region by epigenetic mechanisms in osteoporosis of nonosteoporotic postmenopausal women [45]. These findings require further investigation in terms of the kinetics of SOST and DKK-1 in human patients with RA.

We identified that increase on the serum SOST levels correlated with high FRAX scores of the 10-year risk of major and hip osteoporotic fractures. We did not find other published studies that assessed the correlation between SOST levels and FRAX scores; therefore, our findings should be corroborated by future studies. It has been proposed by some authors that the paradoxical high serum levels of SOST in some patients with osteoporosis may be associated with a feedback loop in response to the loss of bone mass [16, 44]. In consequence, we can hypothesize that this increase in serum SOST levels can be an adaptive response to the osteoporosis observed in our patients, and future studies should be performed to determinate whether these levels constituted a risk factor for osteoporotic fractures. Similar to our findings, Arasu et al. identified in women without rheumatic diseases that high serum levels of SOST and a higher FRAX score, thus pointing-out to a higher risk of hip fractures [44].

One of the main strengths of this study was the inclusion of a broader spectrum of potential bone turnover biomarkers compared to other studies. Moreover, this study included the assessment of the correlation between these molecules with the FRAX scores of the 10-year risk of a major osteoporotic fractures and 10-year probability of a hip fracture. We identified that both sRANKL levels and sRANKL/OPG ratio can be used as biomarkers of osteoporosis, and an association with the estimated 10-years risk to the major osteoporotic and hip fracture assessed by FRAX in patients with RA was found. However, other molecules, such as DKK-1 and SOST, require further investigation to support our results in RA patients.

Nevertheless, several limitations of our study should be considered. Our study included RA patients with a long disease duration, and the characteristics of marker levels could be affected by disease duration. Therefore, further studies should include nontreated patients with early RA. In addition, considering the cross-sectional design of this study, further longitudinal studies should be performed to identify if these patients with higher serum levels of sRANKL or SOST have a major incidence of fractures compared to patients with normal levels. Although FRAX is a useful tool for predicting the risk of major osteoporotic fractures or hip fracture in RA, many patients can develop osteoporotic fractures even with low FRAX scores. Therefore, the utility of these biomarkers, mainly sRANKL levels and sRANKL/OPG ratio, can be considered potential clinical tools that help predict future fractures in RA patients.

## 5. Conclusions

This study identifies that high sRANKL levels and the sRANKL/OPG ratio can be useful to detect a subgroup of patients with RA who had an increase in the 10-year risk of a major osteoporotic fracture and the 10-year risk of a hip fracture. High levels of sRANKL and an elevated sRANKL/OPG ratio were associated with osteoporosis. In contrast, SOST correlated negatively with the risk fractures assessed by FRAX. The determination of the sRANKL and sRANKL/OPG ratio can complement the findings of the FRAX score by predicting a clinical subgroup of patients with a high risk of developing osteoporotic fractures. We propose that the measurement of these molecules can contribute to identify RA patients at high risk of osteoporotic fractures by allowing clinicians to focus on the decision-making process when treating this high-risk group of patients.

## Figures and Tables

**Table 1:** Comparison of selected patient characteristics between the groups with rheumatoid arthritis with osteoporosis vs. without osteoporosis.

Clinical features	RA+ osteoporosis *n* = 77	RA without osteoporosis *n* = 79	*p*
Females, *n* (%)	77 (100)	79 (100)	—
Age (yr), mean ± SD	63 ± 9	56 ± 10	<0.001
Body mass index (kg/m^2^), mean ± SD	26.6 ± 4.5	29.2 ± 4.3	<0.001
Disease characteristics			
Disease duration (yr), mean ± SD	14 ± 10	11 ± 9	0.058
Menopausal duration (yr), mean ± SD	17 ± 10	10 ± 9	<0.001
DAS28 score, mean ± SD	3.7 ± 1.4	3.3 ± 1.4	0.086
HAQ-Di score, mean ± SD	0.52 ± 0.51	0.41 ± 0.56	0.173
ESR (mm/hr), mean ± SD	28 ± 11	23 ± 10	0.006
Rheumatoid factor (UI/mL), mean ± SD	157.6 ± 489.5	81.7 ± 135.8	0.186
Tumor necrosis factor-*α* (pg/mL), mean ± SD	22.9 ± 38.8	26.3 ± 84.2	0.711
ACPAs (+), *n* (%)	59 (76.6)	53 (67.1)	0.215
Anti-CCP2 (+), *n* (%)	45 (58.4)	47 (59.5)	1.000
Anti-CCP2 (RU/mL), mean ± SD	92.6 ± 112.2	95.7 ± 124.3	0.867
Anti-MCV (+), *n* (%)	51 (66.2)	40 (50.6)	0.053
Anti-MCV (U/mL), mean ± SD	231.8 ± 313.3	185.1 ± 317.2	0.356
Treatment characteristics			
cs-DMARD use, *n* (%)	62 (80.5)	63 (79.7)	1.000
Monotherapy (1 cs-DMARD), *n* (%)	27 (43.5)	30 (47.6)	0.721
Politherapy (2 cs-DMARD or more), *n* (%)	33 (52.4)	35 (56.5)	0.721
Glucocorticoid use, *n* (%)	64 (83.1)	62 (78.5)	0.544
Glucocorticoid dose (mg/day), mean ± SD	5.9 ± 6.9	4.8 ± 4.8	0.241
Biomarker serum levels			
sRANKL (pmol/L), mean ± SD	1063.7 ± 1257.1	602.3 ± 634.1	0.005
OPG (pg/mL), mean ± SD	113.8 ± 85.4	119.2 ± 89.4	0.670
sRANKL/OPG ratio	14.1 ± 21.6	7.6 ± 9.1	0.017
DKK-1 (pg/mL), mean ± SD	204.1 ± 191.8	279.4 ± 230.2	0.028
SOST (pg/mL), mean ± SD	114.1 ± 56.2	163.5 ± 102.9	<0.001
OC (ng/mL), mean ± SD	4.8 ± 4.6	5.9 ± 5.9	0.190
CTSK (pg/mL), mean ± SD	3.1 ± 1.6	2.9 ± 1.3	0.494

DAS28: Disease Activity Score for 28 joints; HAQ-DI: Health Assessment Questionnaire Disability Index; BMD: bone mineral density; ESR: erythrocyte sedimentation rate; ACPAs: antibodies against cyclic-citrullinated peptides/proteins included anti-CCP2 or anti-MCV; cs-DMARD: conventional synthetic-disease-modifying antirheumatic drugs; sRANKL: Soluble Receptor Activator for Nuclear Factor kappa B Ligand; OPG: osteoprotegerin; DKK-1: Dickkopf-1; SOST: sclerostin; OC: osteocalcin; CTSK: cathepsin k. Qualitative variables were expressed in frequencies (%) and quantitative variables in means ± standard deviations (SD). Statistical tests: Chi-square test (or Fisher exact test if applicable) for comparisons between proportions. Independent sample Student *t*-tests were conducted for comparisons between means, and *p* values were obtained comparing RA patients with osteoporosis vs. RA patients without osteoporosis. The group of patients without osteoporosis included patients with osteopenia or with normal BMD (*T* − score higher than − 2.5 SD).

**Table 2:** Correlations of bone mineral density in two regions in RA patients with clinical characteristics, serological features, and bone biomarker serum levels.

	Total hip BMD (g/cm^2^)		Lumbar spine BMD (g/cm^2^)	
	*r*	*p*	*r*	*p*
Clinical characteristics and serological features				
Age (yr)	-0.462	<0.001	-0.380	<0.001
Body mass index (kg/cm^2^)	0.275	0.001	0.179	0.025
Disease duration (yr)	-0.234	0.003	-0.015	0.852
Menopausal duration (yr)	-0.385	<0.001	-0.348	<0.001
DAS28 score	-0.121	0.133	0.003	0.970
HAQ-Di score	-0.058	0.473	-0.026	0.751
ESR (mm/hr)	-0.236	0.003	-0.103	0.202
Rheumatoid factor (UI/mL)	-0.101	0.210	-0.052	0.518
Tumor necrosis factor-*α* (pg/mL)	0.025	0.756	0.076	0.348
Anti-CCP2 (RU/mL)	-0.053	0.510	-0.064	0.426
Anti-MCV (U/mL)	-0.154	0.055	-0.012	0.880
Treatment characteristics				
Glucocorticoid dose (mg)	-0.031	0.697	-0.108	0.180
Biomarker serum levels				
sRANKL (pmol/L)	-0.267	0.001	-0.044	0.587
OPG (pg/mL)	0.007	0.933	0.077	0.338
sRANKL/OPG ratio	-0.222	0.005	-0.043	0.594
DKK-1(pg/mL)	0.209	0.009	0.157	0.050
SOST (pg/mL)	0.317	<0.001	0.370	<0.001
OC (ng/mL)	0.085	0.294	0.085	0.292
CTSK (pg/mL)	-0.006	0.945	-0.125	0.121

BMD: bone mineral density; DAS28: Disease Activity Score for 28 joints; HAQ-DI: Health Assessment Questionnaire Disability Index; ESR: erythrocyte sedimentation rate; anti-CCP2: 2nd-generation antibodies against citrullinated proteins; anti-MCV: antimutated citrullinated vimentin antibodies; sRANKL: Soluble Receptor Activator for Nuclear Factor kappa B Ligand; OPG: osteoprotegerin; DKK-1: Dickkopf-1; SOST: sclerostin; OC: osteocalcin; CTSK: cathepsin k. Correlations between biomarker serum levels and quantitative variables were performed using Pearson test.

**Table 3:** Correlations of estimated in 10-year of major osteoporosis fracture risk, hip fracture risk (FRAX score), and biomarker serum levels.

	Estimated 10-year of major osteoporosis fracture risk (FRAX score)		Estimated 10-year of hip fracture risk (FRAX score)	
	*r*	*p*	*r*	*p*
Clinical characteristics and serological features				
RA disease duration (yr)	0.230	0.004	0.225	0.005
Menopausal duration (yr)	0.431	<0.001	0.306	<0.001
DAS28 score	-0.003	0.975	0.015	0.857
HAQ-Di score	-0.023	0.777	-0.042	0.607
ESR (mm/hr)	0.261	0.001	0.243	0.002
Rheumatoid factor (UI/mL)	0.061	0.453	0.071	0.377
Tumor necrosis factor-*α* (pg/mL)	-0.075	0.350	-0.052	0.521
Anti-CCP2 (RU/mL)	0.038	0.641	-0.012	0.882
Anti-MCV (U/mL)	0.082	0.310	0.104	0.198
Treatment characteristics				
Glucocorticoid dose (mg)	-0.013	0.876	-0.016	0.838
Biomarker serum levels				
Serum sRANKL levels (pmol/L)	0.237	0.003	0.244	0.002
Serum OPG levels (pg/mL)	-0.012	0.881	-0.006	0.942
sRANKL/OPG ratio	0.208	0.009	0.217	0.006
Serum DKK-1 levels (pg/mL)	-0.131	0.104	-0.110	0.173
Serum SOST levels (pg/mL)	-0.240	0.003	-0.209	0.009
Serum OC levels (ng/mL)	-0.059	0.468	-0.029	0.720
Serum CTSK levels (pg/mL)	-0.015	0.848	-0.053	0.515

FRAX: fracture risk assessment tool; DAS28: Disease Activity Score for 28 joints; HAQ-DI: Health Assessment Questionnaire Disability Index; ESR: erythrocyte sedimentation rate; ACPAs: antibodies against cyclic-citrullinated peptides/proteins included anti-CCP2 or anti-MCV; sRANKL: Soluble Receptor Activator for Nuclear Factor kappa B Ligand; OPG: osteoprotegerin; DKK-1: Dickkopf-1; SOST: sclerostin; OC: osteocalcin; CTSK: cathepsin k. Correlations between biomarker serum levels and FRAX indices were performed using Pearson test.

## Data Availability

The datasets generated during this study are available from corresponding authors on responsible request.

## Supplementary Materials

Supplementary Materials Supplementary Table 1 shows the comparison between groups of patients divided by the severity of the FRAX score for major osteoporotic fractures. Higher sRANKL levels were observed in the group with a 10-year risk of major osteoporotic fractures > 20%, compared with the group with a 10-year risk of major osteoporotic fractures between 10 and 20% and the group with a 10-year risk of major osteoporotic fractures < 10% (*p* = 0.001). Similarly, the higher sRANKL/OPG ratio was observed in the group with a 10-year risk of major osteoporotic fractures > 20%, compared to other two groups (*p* = 0.001). Instead, the lower serum concentrations of SOST were observed in the group with a 10-year risk of major osteoporotic fractures > 20%, compared with the other groups (*p* = 0.01). No statistical differences were observed in the other biomarker levels between these three groups. Supplementary Table 2 shows the comparison between groups of RA patients divided by the severity of the FRAX score for hip fracture. Higher sRANKL levels were observed in the group with a 10-year risk of hip fracture > 10%, compared with the group of a 10-year risk of hip fracture between 3 and 10% and the group of a 10-year risk of hip fracture < 3% (*p* < 0.001). Similarly, the higher sRANKL/OPG ratio was observed in the group with a 10-year risk of hip fracture > 10%, compared to other two groups (*p* < 0.001). Instead, lower serum concentrations of SOST were observed in the group with a 10-year risk of hip fracture > 10%, compared with the other groups (*p* = 0.016). We did not observe significant statistical differences in the other biomarker levels between these three groups.
