# Supplementary material for: Assessment of Serum sRANKL, sRANKL/OPG Ratio, and Other Bone Turnover Markers with the Estimated 10-Year Risk of Major and Hip Osteoporotic Fractures in Rheumatoid Arthritis: A Cross-Sectional Study
Source: Biomed Res Int. 2021 Aug 30;2021:5567666. doi: 10.1155/2021/5567666 (PMC8421166; doi:10.1155/2021/5567666)
Supplement: Supplementary Materials — Supplementary Table 1 shows the comparison between groups of patients divided by the severity of the FRAX score for major osteoporotic fractures. Higher sRANKL levels were observed in the group with a 10-year risk of major osteoporotic fractures > 20%, compared with the group with a 10-year risk of major osteoporotic fractures between 10 and 20% and the group with a 10-year risk of major osteoporotic fractures < 10% (p = 0.001). Similarly, the higher sRANKL/OPG ratio was observed in the group with a 10-year risk of major osteoporotic fractures > 20%, compared to other two groups (p = 0.001). Instead, the lower serum concentrations of SOST were observed in the group with a 10-year risk of major osteoporotic fractures > 20%, compared with the other groups (p = 0.01). No statistical differences were observed in the other biomarker levels between these three groups. Supplementary Table 2 shows the comparison between groups of RA patients divided by the severity of the FRAX score for hip fracture. Higher sRANKL levels were observed in the group with a 10-year risk of hip fracture > 10%, compared with the group of a 10-year risk of hip fracture between 3 and 10% and the group of a 10-year risk of hip fracture < 3% (p < 0.001). Similarly, the higher sRANKL/OPG ratio was observed in the group with a 10-year risk of hip fracture > 10%, compared to other two groups (p < 0.001). Instead, lower serum concentrations of SOST were observed in the group with a 10-year risk of hip fracture > 10%, compared with the other groups (p = 0.016). We did not observe significant statistical differences in the other biomarker levels between these three groups. [file 5567666.f1.docx]

**Supplementary:**

Supplementary Table 1 shows the comparison between groups of patients divided by the severity of the FRAX score for major osteoporotic fractures. Higher sRANKL levels were observed in the group with a 10-year risk of major osteoporotic fractures>20%, compared with the group with a 10-year risk of major osteoporotic fractures between 10-20%, and the group with a10-year risk of major osteoporotic fractures <10% (p=0.001). Similarly, the higher sRANKL/OPG ratio was observed in the group with a 10-year risk of major osteoporotic fractures >20%, compared to other two groups (p=0.001). Instead, the lower serum concentrations of SOST was observed in the group with a 10-year risk of major osteoporotic fractures >20%, compared with the other groups (p=0.01). No statistical differences were observed in the other biomarkers levels between these three groups.

**Supplementary table 1.** Comparison of the serum bone turnover markers levels between groups of RA patients divided by their 10-year risk groupsof major osteoporotic fracturesin RA patients.

| **Serum molecules** | **10-year probability of major osteoporotic fractures** | | | |
| --- | --- | --- | --- | --- |
|  | **High**  **(>20%)**  **n = 20** | **Moderate**  **(10–20%)**  **n = 43** | **Low**  **(<10%)**  **n = 93** | ***p-value*** |
| sRANKL (pmol/L)*, mean ± SD*$\mathrm{mean}\mathrm{SD}$ | **1570.4 ± 1770.1** | **860.6 ± 1028.5** | **656.7 ± 675.8** | **0.001^a,b^** |
| OPG (pg/mL)*, mean ± SD*$\mathrm{mean}\mathrm{SD}$ | 110.5 ± 57.1 | 119.4 ± 70.6 | 116.5 ± 85.8 | 0.916 |
| sRANKL/OPG ratio | **23.3 ± 34.5** | **10.2 ± 14.0** | **8.5 ± 9.9** | **0.001^a,b^** |
| DKK-1 (pg/mL)*, mean ± SD*$\mathrm{mean}\mathrm{SD}$ | 217.4 ± 188.5 | 201.2 ± 185.9 | 266.5 ± 230.4 | 0.222 |
| SOST (pg/mL)*, mean ± SD*$\mathrm{mean}\mathrm{SD}$ | **106.3 ± 53.8** | **117.8 ± 78.1** | **156.0 ± 92.2** | **0.010 ^c^** |
| OC (ng/mL)*, mean ± SD*$\mathrm{mean}\mathrm{SD}$ | 4.4 ± 2.9 | 5.3 ± 5.3 | 5.7 ± 5.7 | 0.633 |
| CTSK (pg/mL)*, mean ± SD*$\mathrm{mean}\mathrm{SD}$ | 2.7 ± 1.1 | 3.3 ± 1.8 | 2.9 ± 1.5 | 0.166 |

FRAX: Fracture Risk Assessment Tool; sRANKL: Soluble Receptor Activator for Nuclear Factor kappa B Ligand; OPG: Osteoprotegerin; DKK-1: Dickkopf-1; SOST: Sclerostin; OC: Osteocalcin; CTSK: Cathepsin k. Quatitative variables were expressed in means ± Standard Deviations (SD). Statistical tests: Independent sample ANOVA one-way test were conducted for comparisons between means, and *p* values were obtained comparing RA patients with high (>20% FRAX score), moderate (10 to 20% FRAX score) and low risk (<10% FRAX score) in the 10-year probability of major osteoporotic fractures; and comparing RA patients with high (>10% FRAX score), moderate (3 to 10% FRAX score) and low risk (<3% FRAX score) in the 10-year probability of hip fracture by FRAX score. Bonferroni post-hoc test was used.

^a^ High versus low risk of major osteoporotic fractures (significance of p≤0.05).

^b^ High versus moderate risk of major osteoporotic fractures (significance of p≤0.05).

^c^ Moderate versus low risk of major osteoporotic fractures (significance of p≤0.05).

Supplementary Table 2 shows the comparison between groups of RA patients divided by the severity of the FRAX score for hip fracture. Higher sRANKL levels were observed in the group with a 10-year risk of hip fracture >10%, compared with the group of a 10-year risk of hip fracture between 3-10%, and the group of a 10-year risk of hip fracture <3% (p<0.001). Similarly, the higher sRANKL/OPG ratio was observed in the group with a 10-year risk of hip fracture >10%, compared to other two groups (p<0.001). Instead, lower serum concentrations of SOST were observed in the group with a 10-year risk of hip fracture >10%, compared with the other groups (p=0.016). We did not observe significant statistical differences in the other biomarkers levels between these three groups.

**Supplementary table 2.** Comparison of the serum bone turnover markers levels between groups of RA patients divided by their 10-year risk groupsof hip fractures in RA patients.

| **Serum molecules** | **10-year probability of hip fractures** | | | |
| --- | --- | --- | --- | --- |
|  | **High**  **(>10%)**  **n = 17** | **Moderate**  **(3–10%)**  **n = 37** | **High**  **(<3%)**  **n = 102** | ***p-value*** |
| sRANKL (pmol/L)*, mean ± SD*$\mathrm{mean}\mathrm{SD}$ | **1746.9 ± 1847.6** | **784.2 ± 925.6** | **693.8 ± 758.1** | **<0.001^a,b^** |
| OPG (pg/mL)*, mean ± SD*$\mathrm{mean}\mathrm{SD}$ | 101.4 ± 52.1 | 124.5 ± 74.9 | 116.5 ± 83.1 | 0.601 |
| sRANKL/OPG ratio | **27.0 ± 36.2** | **8.3 ± 9.2** | **9.1 ± 11.9** | **<0.001^a,b^** |
| DKK-1 (pg/mL)*, mean ± SD*$\mathrm{mean}\mathrm{SD}$ | 205.9 ± 166.7 | 202.4 ± 200.8 | 262.7 ± 225.4 | 0.262 |
| SOST (pg/mL)*, mean ± SD*$\mathrm{mean}\mathrm{SD}$ | **107.2 ± 51.6** | **114.4 ± 80.3** | **153.4 ± 90.4** | **0.016 ^c^** |
| OC (ng/mL)*, mean ± SD*$\mathrm{mean}\mathrm{SD}$ | 4.6 ± 2.9 | 5.3 ± 5.9 | 5.5 ± 5.5 | 0.808 |
| CTSK (pg/mL)*, mean ± SD*$\mathrm{mean}\mathrm{SD}$ | 2.4 ± 0.9 | 3.1 ± 1.5 | 3.0 ± 1.5 | 0.252 |

FRAX: Fracture Risk Assessment Tool; DAS28: Disease Activity Score for 28 joints; HAQ-DI: Health Assessment Questionnaire-Disability Index; ESR: erythrocyte sedimentation rate; ACPAs: antibodies against cyclic-citrullinated peptides/proteins included Anti-CCP2 or Anti-MCV; sRANKL: Soluble Receptor Activator for Nuclear Factor kappa B Ligand; OPG: Osteoprotegerin; DKK-1: Dickkopf-1; SOST: Sclerostin; OC: Osteocalcin; CTSK: Cathepsin k. Qualitative variables were expressed in frequencies (%), and quantitative variables in means ± Standard Deviations (SD). Statistical tests: Chi-square test (or Fisher exact test if applicable) for comparisons between proportions; Independent sample ANOVA one-way test were conducted for comparisons between means, and *p* values were obtained comparing RA patients with high (>20% FRAX score), moderate (10 to 20% FRAX score) and low risk (<10% FRAX score) in the 10-year probability of major osteoporotic fractures; and comparing RA patients with high (>10% FRAX score), moderate (3 to 10% FRAX score) and low risk (<3% FRAX score) in the 10-year probability of hip fracture by FRAX score. Bonferroni post-hoc test was used.

^a^ High versus low risk of major osteoporotic fractures (significance of p≤0.05).

^b^ High versus moderate risk of major osteoporotic fractures (significance of p≤0.05).

^c^ Moderate versus low risk of major osteoporotic fractures (significance of p≤0.05).
